# Supplementary material for: A FRET sensor of C-terminal movement reveals VRAC activation by plasma membrane DAG signaling rather than ionic strength
Source: eLife. 2019 Jun 18;8:e45421. doi: 10.7554/eLife.45421 (PMC6597245; doi:10.7554/eLife.45421)
Supplement: Figure 1—source data 1. — The statistics in the Tables accompany data in Figure 1C,E and F. Change of CFP intensity [%] (Figure 1C). [file elife-45421-fig1-data1.docx]

Figure 1–source data 1. Statistics of acceptor-bleaching experiment and hypotonicity-induced FRET changes. The statistics in the Tables accompany data in Figure 1C, E and F.

Change of CFP intensity [%] (Figure 1C):

|  | A-CFP/A-YFP | | A-CFP/E-YFP | | A-YFP/E-CFP | |
| --- | --- | --- | --- | --- | --- | --- |
|  | bleached | control | bleached | control | bleached | control |
| mean: | 29.6 | 7.3 | 14.4 | -4.0 | 31.3 | 12.3 |
| s.e.m.: | 3.0 | 2.8 | 3.1 | 2.0 | 4.7 | 2.6 |
| n (cells): | 11 | 7 | 11 | 6 | 5 | 5 |
| p: | 0.00012 | | 0.00036 | | 0.02 | |

|  | A-CFP/CD4-YFP | | A-CFP/E-YFP (ER) | | A-CFP/CD4-YFP(ER) | |
| --- | --- | --- | --- | --- | --- | --- |
|  | bleached | control | bleached | control | bleached | control |
| mean: | -4.3 | -6.9 | 14.1 | -5.6 | -6.1 | -7.4 |
| s.e.m.: | 1.5 | 3.7 | 4.0 | 2.4 | 1.3 | 0.4 |
| n (cells): | 8 | 4 | 5 | 5 | 6 | 3 |
| p: | 0.60 | | 0.007 | | 0.44 | |

Normalized cFRET (Figure 1E):

|  | A-CFP/  E-YFP (HeLa) | A-CFP/  E-YFP (HEK) | A-CFP/  A-YFP | GluA2-6Y-10C | CFP-18aa-YFP |
| --- | --- | --- | --- | --- | --- |
| mean: | 0.95 | 0.90 | 0.94 | 0.99 | 1.02 |
| s.e.m.: | 0.01 | 0.01 | 0.01 | 0.01 | 0.01 |
| *cells:* | *47* | *29* | *24* | *13* | *11* |
| n (dishes): | 9 | 8 | 8 | 4 | 4 |
| p (vs. Iso) | 0.00011 | 7x10^-5^ | 0.00025 | 0.47 | 0.16 |

Normalized cFRET (Figure 1G):

| mOsm: | 50 | 100 | 150 | 200 | 250 | 340 | 400 |
| --- | --- | --- | --- | --- | --- | --- | --- |
| mean: | 0.76 | 0.80 | 0.83 | 0.90 | 0.95 | 1.00 | 1.03 |
| s.e.m.: | 0.02 | 0.01 | 0.02 | 0.02 | 0.01 | 0.0001 | 0.006 |
| n (dishes): | 11 | 11 | 11 | 7 | 11 | 21 | 7 |
