# Supplementary material for: A FRET sensor of C-terminal movement reveals VRAC activation by plasma membrane DAG signaling rather than ionic strength
Source: eLife. 2019 Jun 18;8:e45421. doi: 10.7554/eLife.45421 (PMC6597245; doi:10.7554/eLife.45421)
Supplement: Figure 2—source data 1. — The statistics in the Table accompany data in Figure 2D. Normalized cFRET (Figure 2D). [file elife-45421-fig2-data1.docx]

Figure 2–source data 1. Statistics of hypotonicity-induced FRET changes. The statistics in the Table accompany data in Figure 2D.

Normalized cFRET (Figure 2D):

|  | ER | noGlyc PM |
| --- | --- | --- |
| mean: | 1.00 | 0.93 |
| s.e.m.: | 0.01 | 0.01 |
| *cells:* | *23* | *21* |
| n (dishes): | 16 | 7 |
| p (vs. Iso) | 0.8 | 0.00019 |
