# Supplementary material for: A FRET sensor of C-terminal movement reveals VRAC activation by plasma membrane DAG signaling rather than ionic strength
Source: eLife. 2019 Jun 18;8:e45421. doi: 10.7554/eLife.45421 (PMC6597245; doi:10.7554/eLife.45421)
Supplement: Figure 3—source data 1. — The statistics in the Tables accompany data in Figure 3C, Figure 3—figure supplement 2B and C. Normalized cFRET (Figure 3C). [file elife-45421-fig3-data1.docx]

Figure 3–source data 1. Statistics of hypotonicity-induced FRET changes and cholesterol depletion. The statistics in the Tables accompany data in Figure 3C, Figure 3–figure supplement 2B and C.

Normalized cFRET (Figure 3C):

|  | ER | Golgi | PM |
| --- | --- | --- | --- |
| mean: | 0.99 | 0.98 | 0.94 |
| s.e.m.: | 0.01 | 0.01 | 0.01 |
| *cells:* | *10* | *26* | *11* |
| n (dishes): | 4 | 5 | 5 |
| p (vs. Iso) | 0.7 | 0.2 | 0.00034 |

Normalized cFRET (Figure 3–figure supplement 2B):

|  | untreated | LatB | MbCD |
| --- | --- | --- | --- |
| mean: | 0.95 | 0.93 | 0.88 |
| s.e.m.: | 0.006 | 0.008 | 0.01 |
| *cells:* | *47* | *26* | *46* |
| n (dishes): | 9 | 8 | 10 |
| p (vs. untreated) | - | 0.9 | 0.0006 |

Fluorescence intensity [a.u.] (Figure 3–figure supplement 2C):

|  | untreated | MbCD |
| --- | --- | --- |
| mean: | 9695 | 7792 |
| s.e.m.: | 570 | 129 |
| *cells:* | *>300* | *>300* |
| n (dishes): | 5 | 5 |
| p (vs. untreated) | - | 0.04 |
