# Supplementary material for: A FRET sensor of C-terminal movement reveals VRAC activation by plasma membrane DAG signaling rather than ionic strength
Source: eLife. 2019 Jun 18;8:e45421. doi: 10.7554/eLife.45421 (PMC6597245; doi:10.7554/eLife.45421)
Supplement: Figure 4—source data 1. — The statistics in the Tables accompany data in Figure 4C, Figure 4—figure supplement 1C and Figure 4—figure supplement 2A. Currents [nA] or current densities [pA/pF] (Figure 4C). [file elife-45421-fig4-data1.docx]

Figure 4–source data 1. Statistics of currents and FRET changes in presence of PMA or Gö6983. The statistics in the Tables accompany data in Figure 4C, Figure 4–figure supplement 1C and Figure 4–figure supplement 2A.

Currents [nA] or current densities [pA/pF] (Figure 4C):

|  | A-CFP/E-YFP  (current [nA]) | | | endogenous VRAC  (current density [pA/pF]) | | |
| --- | --- | --- | --- | --- | --- | --- |
|  | Iso | Iso, PMA | untransf., PMA | Iso | Hypo | PMA |
| mean: | -0.15 | -0.41 | -0.17 | -3.8 | -14.4 | -16.7 |
| s.e.m.: | 0.03 | 0.11 | 0.19 | 1.1 | 3.9 | 5.8 |
| n (cells): | 9 | 8 | 8 | 7 | 7 | 8 |

Normalized cFRET (Figure 4–figure supplement 1C):

|  | A-CFP-FM_2_/E-YFP | | |
| --- | --- | --- | --- |
|  | ER | Golgi | PM |
| mean: | 0.99 | 0.99 | 0.94 |
| s.e.m.: | 0.01 | 0.02 | 0.01 |
| *cells:* | *5* | *12* | *6* |
| n (dishes): | 4 | 5 | 5 |
| p (vs. Iso) | 0.77 | 0.78 | 0.02 |

Normalized cFRET (Figure 4–figure supplement 2A):

|  | A-CFP/E-YFP | | |
| --- | --- | --- | --- |
|  | Hypo | Iso Gö | Hypo Gö |
| mean: | 0.93 | 1.16 | 1.39 |
| s.e.m.: | 0.02 | 0.06 | 0.11 |
| n (cells): | 7 | | |
