# Supplementary material for: A FRET sensor of C-terminal movement reveals VRAC activation by plasma membrane DAG signaling rather than ionic strength
Source: eLife. 2019 Jun 18;8:e45421. doi: 10.7554/eLife.45421 (PMC6597245; doi:10.7554/eLife.45421)
Supplement: Figure 5—source data 1. — The statistics in the Tables accompany data in Figure 5. Currents [nA] (Figure 5A). [file elife-45421-fig5-data1.docx]

Figure 5–source data 1. Statistics of currents and FRET changes in presence of CRT 0066101 or DOG. The statistics in the Tables accompany data in Figure 5.

Currents [nA] (Figure 5A):

|  | Hypo | Hypo, CRT0066101 |
| --- | --- | --- |
| mean: | -0.26 | -0.07 |
| s.e.m.: | 0.06 | 0.02 |
| n (cells): | 4 | 5 |
| p: | 0.048 | |

Currents [nA] (Figure 5B):

|  | Iso | Hypo | Hypo, CRT0066101 | Hypo,  Gö6983 |
| --- | --- | --- | --- | --- |
| mean: | -0.15 | -0.70 | -0.33 | -0.83 |
| s.e.m.: | 0.03 | 0.32 | 0.04 | 0.13 |
| n (cells): | 9 | 7 | 4 | 5 |

Current recovery [%] (Figure 5C):

|  | A-CFP/E-YFP | | endogenous VRAC | |
| --- | --- | --- | --- | --- |
|  | untreated | DOG | untreated | DOG |
| mean: | 87.8 | 27.9 | 54.7 | 18.3 |
| s.e.m.: | 8.7 | 17.4 | 3.8 | 11.1 |
| n (cells): | 3 | 3 | 3 | 4 |
| p: | 0.037 | | 0.034 | |

Normalized cFRET or normalized FRET/Cerulian (Figure 5D):

|  | VRAC activity | | Ionic strength | |
| --- | --- | --- | --- | --- |
|  | Hypo DOG | Iso DOG | Hypo DOG | Iso DOG |
| mean: | 0.90 | 0.94 | 1.10 | 0.98 |
| s.e.m.: | 0.03 | 0.02 | 0.01 | 0.00 |
| *cells:* | *9* | | *23* | |
| n (dishes): | 7 | | 6 | |
| p: | 0.345 | | 6x10^-6^ | |
